# Dietary Supplements Use among Serbian Undergraduate Students of Different Academic Fields

**DOI:** 10.3390/ijerph191711036

**Published:** 2022-09-03

**Authors:** Bojana Vidović, Bojana Đuričić, Marina Odalović, Andrijana Milošević Georgiev, Ivana Tadić

**Affiliations:** 1 Department of Bromatology, Faculty of Pharmacy, University of Belgrade, Vojvode Stepe 450, 11221 Belgrade, Serbia; 2 Department of Social Pharmacy and Pharmaceutical Legislation, Faculty of Pharmacy, University of Belgrade, Vojvode Stepe 450, 11221 Belgrade, Serbia

**Keywords:** dietary supplements, university, students, prevalence, knowledge

## Abstract

The rising popularity of dietary supplements as a part of self-care practice increases interest in monitoring their usage in the general and specific population groups. This study investigated the prevalence and patterns of dietary supplement use among Belgrade University undergraduate students and its variations across different academic study fields. Of the 914 online survey students, 55.7% used dietary supplements during the past year. Female gender, eating behavior, and academic field were significant predictors of dietary supplement use. For all students, the most commonly used dietary supplements were vitamins and minerals, alone or in combination. Magnesium, vitamin C, and B vitamins were the most frequently supplemented micronutrients. The reasons for using, place of purchase, and source of information regarding dietary supplements significantly varied among students of different fields of study. Adverse effects related to dietary supplement use, including gastrointestinal symptoms, skin flushing, dizziness, and heart palpitation, were reported in 4.5% of students. Insufficient knowledge about these products was self-reported by 16.5% of users, more common among non-medical students. Thus, public health interventions are needed to improve students’ knowledge regarding rational and safe dietary supplement use.

## 1. Introduction

Despite rising popularity, there is a lack of global consensus on the definition and categorization of products covered by the term “dietary supplements” (DS) [1,2]. According to the European Union (EU) legislation, DSs are foodstuffs intended to supplement the regular diet and present concentrated sources of nutrients or substances with physiological effects, which are marketed in dosage forms (e.g., capsules, tablets, sachets of powders, liquids) [3]. In addition to vitamins and minerals, DSs may contain many other substances, including amino acids, essential fatty acids, fibers, and certain bioactive compounds derived from plants and other natural sources, alone or in combination. 

The main reasons for DS use have changed over time. Namely, the earliest motives for taking DSs to correct nutritional deficiencies and maintain an adequate intake of certain nutrients to support specific physiological functions have been shifted to prevention or treatment of chronic diseases, improvement of overall health, and enhancement of the quality of life [4]. However, the evidence-based health benefits of DS consumption are still not well established [5]. Furthermore, the increasing availability and self-administration of DSs in the general population and specific subgroups raises public health concerns regarding their efficacy and safety [6]. In addition to possible excessive intake of vitamins, minerals, and other bioactive substances, the presence of biological and chemical contaminants, including intentionally added unapproved substances, and potential interactions DS with concomitantly using medicines increase the risk of harmful effects [7]. Therefore, widespread usage of DS, supported by aggressive media advertising and the common belief that natural substances have only beneficial health effects, needs continuous monitoring of the patterns and motivation for DS use by healthcare professionals and scientists [8].

More than 50% of adults in the USA use DSs [9]. Across European countries, the prevalence of DS usage varies. Evidence of greater consumption in northern than southern countries [10] indicates the influence of cultural and environmental factors on DS usage [11]. The more frequent users of DSs are women, older adults, and people with a higher level of education, socioeconomic status, and healthier lifestyle habits [12,13,14,15].

Previously published studies revealed that university students are more frequent users of DSs than the general population [16,17] and high school students [8]. In addition, patterns of taking DS use vary between medical sciences and non-medical sciences students [18,19,20]. Furthermore, pharmacy students have more positive attitudes and better knowledge about DS use than medical or dental students [21]. However, several previous studies concluded that the beliefs and attitudes of pharmacy and other health sciences students about DS use are rarely based on medical evidence, highlighting the need for improving their knowledge through curriculum reforms and additional education courses [8,19,20,21,22]. Therefore, insights into the sociodemographic, lifestyle, and academic determinants of DS use among university students could help tailor educational interventions to ensure rational DS use [8,19,20,21,22,23].

There are several reports on the prevalence, knowledge, and attitudes toward DS usage [24] and toward DS types [25,26] in the general Serbian population, young athletes [27], and university undergraduate students [28,29,30]. While Miljkovic et al. (2013) paid attention to the specificity of DS use between male and female students [28], other studies investigated differences in DS usage between medical and non-medical students [29] and the influence of pharmacological education on the usage, attitudes, and perceptions of risks associated with DS among medical students [30]. However, we hypothesized that students’ behavior regarding DS use might vary relating to academic discipline. Considering the gap in the literature data, we have conducted a comparative study to explore the habits related to DS usage among undergraduate students in different academic fields in Belgrade, Serbia.

## 2. Materials and Methods

### 2.1. Participants

A quantitative, cross-sectional, online survey was carried out at the University of Belgrade from April to June 2019. The University of Belgrade is the largest university in Serbia, consisting of 31 faculties. Purposive sampling ensured that students of different academic fields were reached. The study protocol ((No. 1971/2) was reviewed and approved by the Ethics Committee for Human Research of the Faculty of Pharmacy, University of Belgrade. With the support of student representatives, students received a survey invitation via email and social networks, with a definition of DS and a link to the questionnaire form. The inclusion criteria were: access to the internet, an active email address, and the ability to complete an online survey. The survey was voluntary and anonymous. The time needed to complete the survey was around 10 min. Students could stop the survey anytime without saving answers or explaining if they decided to leave the study. No survey item required personal information. The students’ responses were analyzed and presented according to the fields of academic study: medical sciences, social sciences and humanities, technology and engineering sciences, and natural and formal sciences.

### 2.2. Questionnaire Data

The survey questionnaire was created by a combination of questions from previously published studies [17,20,31,32] and current practices in the Republic of Serbia. Survey items were divided into two groups: those related to socio-demographic and lifestyle characteristics and those related to DS usage. The first part of the questionnaire collected general information (which faculty the students attend, year of study, gender, birth year, body weight, body height, smoking status, alcohol consumption, meal regularity, and questions about health status). Body mass index (BMI) was calculated based on self-reported body weight and body height. According to WHO recommendations, students were classified as underweight (<18.50 kg/m^2^), normal weight (18.50–24.99 kg/m^2^), overweight (25.0–29.99 kg/m^2^), and obese (≥30.0 kg/m^2^). At the beginning of the second part of the questionnaire, participants were asked, “Have you consumed any DS in the past year?” While DS non-users were asked to identify why they did not use them, DS users were asked about DS usage. This set of questions requested information on the number, types, and subtypes of DS; frequency, duration, primary reasons for using DS; the purpose of DS use; place of purchase; source of recommendation; knowledge about DS; and adverse effects to DS. The majority of items were close-ended, multiple-choice, or yes/no questions. When the participants completed the survey linked to the Google Form, each survey was sent to a database from which it was downloaded as a Microsoft Excel sheet for further data analysis.

### 2.3. Statistical Analysis

The basic students’ characteristics were analyzed using descriptive statistical analysis. The Chi-squared test of independence was used to test group differences for categorical data [33]. For interval variables, the one-way analysis of variance (ANOVA) with the post hoc Tukey–Kramer test was used to test differences between more than two groups [34]. A univariate analysis was used to analyze univariate associations between potential explanatory variables and DS usage, defined as a dependent variable. A crude odds ratio (cOR) with a 95% confidence interval (CI) was used to explain the association between the explanatory and dependent variables. Variables significantly associated with DS usage in the univariate analysis were included in the multivariate logistic regression model [35]. The impact of independent variables on DS usage was estimated as an adjusted odds ratio (aOR) with 95% CI. The values of *p* < 0.05 have been considered statistically significant in all performed tests. The results analysis has been conducted using Microsoft Office Excel 2007 (Microsoft Corp., Redmond, WA, USA) and Predictive Analytics Software, version 28 (SPSS Inc., Chicago, IL, USA). 

## 3. Results

A total of 914 students completed the questionnaire. The socio-demographic, anthropometric and lifestyle characteristics of the participants are summarized in Table 1.

Overall, 79.1% of students were women. Medical sciences students were in the majority (41.9%), followed by engineering and technology (24.8%), social and humanities (19.1%), and natural and formal sciences students (14.2%). The mean age among students was 23.2 ± 2.2 years (range: 19–35). Only 7.2% of students were chronically ill. The most frequent diseases were endocrine, nutritional, and metabolic diseases (1.8%). Based on self-reported weight and height, most students had a normal BMI. The mean BMI was 22.2 ± 3.2 kg/m^2^ (range: 14.7–38.6). More than two-thirds of students were never smokers. No statistically significant differences in BMI and smoking status were found among students in different academic fields (Table 1). On average, the number of meals consumed by students was 3.1 ± 0.85 (range: 1–7). More than half of the students (52.1%) had three meals daily, while 22.4% had two meals. There were no statistically significant differences in eating behavior related to gender (*p* > 0.05), study years (*p* > 0.05), and academic fields (*p* > 0.05). Regular meals were predominantly had by medical sciences students (72.1%), unlike the natural and formal sciences students (60.8%).

More than half of students reported using DS in the past year. In addition, the prevalence of DS usage was the highest in medical sciences students (*p* < 0.001) (Table 1). The most common reason for taking DSs was to maintain health and well-being. Among the reasons for not using DSs, more than two-thirds of non-users believed that DSs are unnecessary (Table 2).

**Figure 1:**
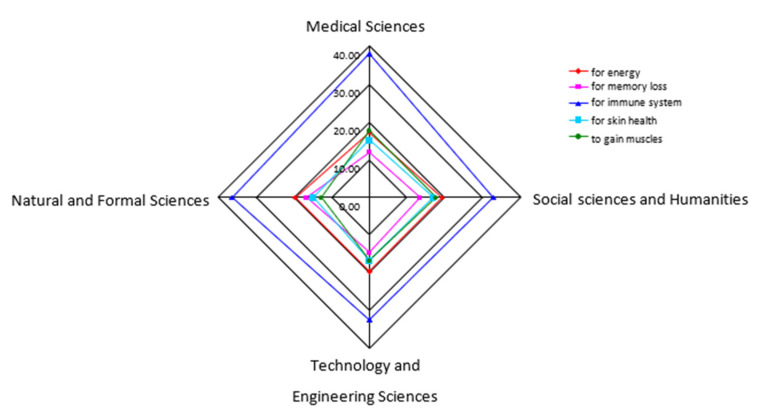
Most common purposes for the use of dietary supplements by students concerning different academic fields (% of students).

The students reported using DSs with a wide range of claimed health effects. However, DSs are commonly used to support the immune system (39.1%), boost energy (20.1%), gain muscles (18.5%), improve skin health (17.3%), and boost memory (14.6%), without a significant difference when comparing students of different scientific fields (*p* > 0.05) (Figure 1).

Associations between DS use and demographic, anthropometric, and lifestyle characteristics are present in Table 3. Despite the significant association in univariate logistic regression analysis, BMI and smoking status were not shown as significant predictors of DS usage in multivariate analysis. However, females were shown to have a 1.5-fold greater chance of using DSs than males. Additionally, students of academic fields other than medical sciences have a 48–75% lower chance of using these products in comparison to medical students. Finally, students with irregular or occasionally regular meals have 36% fewer odds of using DS than students with regular meals (Table 3). 

The most commonly used DSs were vitamins and minerals, alone or in combination, followed by probiotics, proteins/amino acids, fish oils, and herbal supplements. Vitamin C, the B group of vitamins, magnesium, and zinc were the most commonly consumed micronutrients. There were no significant differences regarding the prevalence of the specific types of DS use among students in different academic fields (Table 4).

The mean number of DS usage per student was 1.60 (SD = 1.30, range: 1–4). About one-third of students reported using more than two DSs. The DS usage was most frequent in spring and winter. Most students purchased DSs at pharmacies. Students mentioned a variety of sources of information about DSs. Most students have gathered information on DSs from doctors, followed by pharmacists and friends. Medical sciences students were significantly more likely to consult pharmacists about DSs than other students. However, non-medical sciences students significantly more often used media and friends as sources of information on DSs (Table 5). 

Most students perceived their knowledge about DS as good. Among student groups, medical sciences students had a much better perception of their knowledge than other students (*p* < 0.001) and mostly thought that their knowledge about DS was well (43.3%) or enough (27.9%). Nearly half of students sometimes observed the beneficial effects of DS usage. A small number of students reported adverse reactions due to DS usage, including gastrointestinal symptoms, skin flushing, dizziness, and heart palpitation. There were no differences in reported beneficial and adverse effects rates among students in different academic fields (Table 5).

## 4. Discussion

Our results support findings that the prevalence of DS usage among university students is higher than in the general population. Although data on the overall rate of DS use in Serbia are still limited, one recent study reported that the prevalence of DS use in the general public in northern Serbia was 42.8% [24]. We found that approximately 56% of students used at least one DS in the previous 12 months. This prevalence is less than those previously obtained in students from the University of Niš (68%) in the southeastern part of Serbia [28,29] but comparable with medical students from the University of Kosovska Mitrovica (53%) [30]. However, the prevalence of DS use in our sample was higher than those obtained in students from Croatia (30.5%) [19], Italy (37.4%) [8], and Portugal (46.3%) [36]. In addition to reflecting the global increase in DS consumption [37], observed variations in the prevalence of DS use among different studies may be influenced by differences in demographic, socioeconomic, educational, and lifestyle characteristics of participants and differences in study design. Contrary to our study and an Italian study [8], research in Portugal [36] enrolled participants only from one faculty, and research in Croatia [19] included participants from two faculties. Although there were no differences in gender, nutritional status, or medical and non-medical field distribution, unlike our study, most participants in the Croatian study were junior students [19]. A study among Italian students has reported that the DS rate was significantly higher among older students [8]. Moreover, El Khoury et al. [23] suggested that differences in the prevalence of DS use among students in different countries may be due to underreporting supplementation, existing educational programs targeted at students, and different eating behaviors.

Gender, academic fields, and eating habits were significant predictors of DS usage in this study. The finding that females were significantly more likely to use DSs than males is consistent with previous studies for a general [24] and student population in Serbia [28]. A similar result was obtained by a Portuguese study, where female students consumed more DSs than male students [36], in contrast to a United Arab Emirates (UAE) study, where DS use was significantly higher among male than female students [38]. However, some studies did not find significant gender differences in DS usage [8,18]. There was a significant relationship between academic fields and DS usage, with a higher prevalence of DS use in medical sciences students than in others. This finding is consistent with other studies that reported significantly higher prevalence rates of DS usage among medical students than non-medical students [18,20,29]. Similar trends were also observed by Žeželj et al. [19] and Alhomoud et al. [39]. The evidence suggests that DS users have better dietary patterns, exercise regularly, maintain a healthy weight, and avoid tobacco products [13]. We found that DSs used to be associated with BMI and smoking in students but only in the univariate analysis. Among lifestyle factors, multivariable analyses revealed that eating behavior was the only determinant of DS usage in students. Namely, students who have irregular meals had fewer odds of using DSs. This study also found that most students have used DSs to support general well-being. Bailey et al. [40] reported that DS users were more motivated to use these products for overall health than to fill nutrient gaps in the diet. Therefore, our results support that DS use is associated with adopting more favorable lifestyle choices [13].

Like the Serbian general population [24], the most commonly used types of DSs among university students in this study were vitamins and minerals. These results were consistent with previous studies, which reported that the most popular DSs among students were multivitamins [8,28], vitamin/minerals [18,19,23], and multivitamins/multiminerals [16,38]. In addition, similar to the results of Kobayashi et al.’s [18] research with Japanese college students, we found that individual vitamins and minerals, or multivitamin or multimineral DSs alone, were more popular than multivitamin–mineral combinations. In this study, the most frequently used vitamins and minerals were vitamin C, B vitamins, magnesium, and zinc. Considering approved health claims [41], usage of these subtypes of DSs aligned with the most commonly expected benefits for students, such as immunity support, skin health, and energy and memory boosting. We also observed seasonal variations in DS usage, with the lowest rate (summer/fall) and the highest (winter/spring). Interestingly, while a previous study reported that the usage of vitamins was significantly much higher in medical sciences students than in nonmedical sciences students [19], we did not find significant differences in the type of DS used among students in different academic fields. Alongside vitamins and minerals, probiotics were the most popular type of DS, followed by protein/amino acids and fish oils. Although the result was not statistically significant, a higher prevalence of probiotics used was observed in medical and natural and formal sciences students, implying that formal education contributes to their knowledge regarding probiotics and their health benefits [42]. The protein/amino acids supplement used among students (8.1%) in the present study is in line with previous studies, with a prevalence rate ranging from 7.3% to 17% [16,18,19]. However, a study among Canadian non-athlete students [23] reported that protein supplements had much higher usage rates, ranging between 77.3% for physically active and 85.7% for sedentary DS users. In line with other studies [17,21,43], we found that fish oil, a rich source of long-chain omega-3 fatty acids, is among university students’ most frequently used non-vitamin-mineral supplements. In addition to a general perception that consumption of fish oil DSs is a healthy eating habit [44], there is evidence that omega-3 fatty acids supplementation may have potential anxiolytic benefits for healthy young adults without anxiety disorder diagnosis [45]. 

Although expected to be safe, DS usage without consulting health professionals could increase the risk of adverse effects [32]. In this study, only 52% of all DS users were advised by medical doctors and pharmacists. In contrast, others have listed friends; media sources, including television, radio, print, and the internet; or fitness trainers as sources of recommendation. These findings are similar to a previous study conducted among college students in Italy [8]. In another study, which was conducted in the UAE, the most common source of information for DS users was healthcare providers (30.5%), followed by social media (22.2%) [38]. However, a study in Croatia reported that the internet had the most substantial influence (66.1%) on students’ decisions to take DSs, while healthcare professionals were second (33.2%) [19]. Our study also revealed that social media, including the internet, as an unreliable source of information about DSs, was most popular among students of social sciences and humanities and technology and engineering studies, with rates of about 20%. Additionally, the usage of DSs based on friends’ recommendations was 1.5 to 2 times higher in non-medical than medical sciences students. As expected, the analysis of self-reported knowledge about DS revealed differences among DS users of various types of education. In particular, non-medical sciences students reported having insufficient DS knowledge 2 to 2.5 times more than medical sciences students. At the same time, although medical sciences students are the highest users of DS, they expressed the most negative attitude toward the efficacy of DSs. Namely, as a reason for not using DSs, medical sciences students, more than others, indicated that they do not believe they are necessary. This supports previous findings that their patterns of DS usage are primarily affected by a student’s specific lifestyle rather than education [21]. In addition, medical science students expressed the lowest concern regarding DS safety. However, due to active ingredients, and intentional or nonintentional contaminants, DSs may be linked to public health risks [46]. More than half of the emergency department visits for DS-related adverse events, including cardiac symptoms (palpitations, chest pain, or tachycardia) in the young population, were attributed to weight-loss and energy-boosting DSs [47]. In addition, gastrointestinal symptoms, such as nausea, vomiting, and abdominal pain, are associated with muscle-building supplements [23]. Experiences of side effects among students in this study (4.5%) are consistent with another study on Serbian students [30], but some are lower than in other countries [18,23].

Overall, findings from this study indicate that students’ knowledge about DSs needs to be improved, especially in medical sciences students considering their future professional roles and responsibilities in the healthcare system. Although the pharmacy is the most common place to purchase DSs, previous systematic reviews indicated that pharmacist knowledge about DSs is generally without the actual evidence levels of their efficacy, quality, and safety [48,49]. Therefore, pharmacists and other healthcare professionals need continuous education through curriculum enhancement programs and post-graduation training [50].

There are some strengths and limitations of this study. To date, no published research addressing DS usage among Belgrade university students exists. In addition, the present study’s strength is exploring differences in the prevalence and pattern of DS usage in students in different academic fields. Therefore, the obtained findings could provide a starting point for further research and education strategies to ensure rational and safe DS use. Some limitations of this study include a cross-sectional design, where a cause–effect relationship could not be made. Second, there was an unequal distribution of the study population with an unequal proportion of students in different fields and years of study, and there were more female participants than males. In addition, a limitation of the study is the usage of an online, anonymously filled-in questionnaire on self-reported data regarding DS use from the previous 12 months, which might cause errors in data collection. Finally, further studies need to include students from other universities and consider many more factors as possible predictors of DS usage.

## 5. Conclusions

Our findings highlight that more than half of Serbian undergraduate students use DSs. The high prevalence of DS use among undergraduate students in this study may be due to the majority of them being medical sciences students. That is, medical sciences students are more likely to use DSs than others. The prevalence of DS usage among natural and formal sciences students was 1.5 to 2 times less than among students in other academic fields. At the same time, no significant difference was observed in the DS use between social sciences and humanities students compared to those who study engineering and technology studies. In addition, the predominant participants were female students and students with favorable lifestyle habits, which were reflected by normal weights, regular meals, and never smoking status. In addition to the academic field, gender and eating behavior were associated with DS use among undergraduate students in this study. In addition to the observed variation in the prevalence of DS usage among different academic fields, vitamins and minerals, alone or in combinations, were the most commonly used types of DSs among all student subgroups. However, the reasons for using, place of purchase, and source of information regarding dietary supplements significantly varied among students of different fields of study. Although the self-reported knowledge of medical sciences students was higher than that of other academic fields, they still need to be improved. Incorporating dietary supplements as a topic in medical science curriculums and continuous education courses is important to ensure their own appropriate and safe use of these products and competencies necessary to their future professional roles in the health care system. In addition, the influence of media, the internet, and other unreliable sources of information on the use of DSs, especially among social sciences and humanities students, indicates the need for improving the legislation on the marketing of these products. Overall, the results of this study help to understand DS use practice among undergraduate students, especially non-medical sciences students, suggesting including academic field and other educational factors in future studies dealing with the topic of DS use in this specific population group.

## Figures and Tables

**Table 1:** Sociodemographic, anthropometric, and lifestyle characteristics of students.

Characteristics	Medical Sciences *n* (%)	Social Sciences and Humanities *n* (%)	Technology and Engineering Sciences *n* (%)	Natural and Formal Sciences *n* (%)	All Students N (%)
N (% of all students)	383 (41.9)	174 (19.0)	227 (24.9)	130 (14.2)	914 (100.0)
Gender *					
Female	319 (83.3)	136 (78.2)	164 (72.3)	104 (80.0)	723 (79.1)
Male	64 (16.7)	38 (21.8)	63 (27.8)	26 (20.0)	191 (20.9)
Year of study °^,^**					
1st	24 (6.4)	22 (13.1)	29 (13.2)	20 (15.6)	95 (10.6)
2nd	55 (14.6)	33 (19.6)	31 (14.1)	27 (21.1)	146 (16.4)
3rd	52 (13.8)	39 (23.2)	58 (26.4)	30 (23.4)	179 (20.1)
4th	94 (25.0)	41 (24.4)	68 (30.9)	34 (26.6)	237 (26.6)
5th	100 (26.6)	28 (16.7)	25 (11.4)	14 (10.9)	167 (18.7)
6th	51 (13.6)	5 (2.9)	9 (4.1)	3 (2.3)	68 (7.6)
BMI categories °^,†^					
Underweight	26 (6.9)	11 (6.6)	21 (9.5)	7 (5.5)	65 (7.3)
Normal	292 (77.7)	124 (73.8)	162 (73.3)	96 (75.6)	674 (75.6)
Overweight	51 (13.6)	27 (16.1)	32 (14.5)	18 (14.2)	128 (14.3)
Obese	7 (1.9)	6 (3.6)	6 (2.7)	6 (4.7)	25 (2.8)
Smoking status ^†^					
Current smoker	54 (14.1)	32 (18.4)	41 (18.1)	20 (15.4)	147 (16.1)
Occasional smoker	41 (10.7)	22 (12.6)	24 (10.6)	9 (6.9)	96 (10.5)
Never smoker	272 (71.0)	109 (62.6)	153 (67.4)	96 (73.9)	630 (68.9)
Ex-smoker	16 (4.2)	11 (6.3)	9 (3.9)	5 (3.9)	41 (4.5)
Eating behavior ^†^					
Regular meals	276 (72.1)	122 (70.1)	160 (70.5)	79 (60.8)	637 (69.7)
Occasionally regular meals	60 (15.7)	29 (16.7)	42 (18.5)	30 (23.1)	161 (17.6)
Irregular meals	47 (12.3)	23 (13.2)	25 (11.0)	21 (16.2)	116 (12.7)
Dietary supplement use **					
User	261 (68.2)	88 (50.6)	116 (51.1)	44 (33.9)	509 (55.7)
Not user	122 (31.9)	86 (49.4)	111 (48.9)	86 (66.2)	405 (44.3)

*n*-number of students; BMI-body mass index; ° Data on the year of the study and BMI categories were missing for 22 students. * Statistically significant differences between students of different academic fields (*p* < 0.05). ** Statistically significant differences between students of different academic fields (*p* < 0.001). ^†^ No statistically significant differences in frequencies between students of different scientific fields (Chi-square test).

**Table 2:** Reasons for using/not using dietary supplements among students ^#^.

Reason	Medical Sciences *n* (%)	Social Sciences and Humanities *n* (%)	Technology and Engineering Sciences *n* (%)	Natural and Formal Sciences *n* (%)	Dietary Supplement Users/Not Users (%)
For using *					
Prevention of nutrition deficiencies *	99 (28.7)	18 (15.8)	34 (21.2)	16 (27.1)	167 (24.6)
Disease prevention	39 (11.3)	16 (14.0)	19 (11.9)	7 (11.9)	81 (11.9)
General well-being	200 (58.0)	71 (62.3)	99 (61.9)	33 (55.9)	403 (59.5)
Other	7 (2.0)	9 (7.9)	8 (5.0)	3 (5.1)	27 (4.0)
For not using					
Do not believe they are necessary **	100 (73.0)	57 (53.3)	72 (55.0)	69 (61.1)	298 (61.1)
Not enough information on dietary supplements **	20 (14.6)	26 (24.3)	36 (27.5)	25 (22.1)	107 (21.9)
Do not believe they are safe	14 (10.2)	18 (16.8)	18 (13.7)	15 (13.3)	65 (13.3)
They are too expensive	3 (2.2)	6 (5.6)	5 (3.8)	4 (3.5)	18 (3.7)

^#^ multiple responses; * *p* < 0.05; ** *p* < 0.01.

**Table 3:** Logistic regression analysis of independent predictors of dietary supplement use.

Characteristic	cOR (CI)	aOR (CI)
Gender		
Male	Reference	Reference
Female	1.77 (1.28–2.44) *	1.50 (1.04–2.15) *
Academic field		
Medical sciences	Reference	Reference
Social sciences and Humanities	0.48 (0.33–0.69) **	0.50 (0.34–0.73) **
Technology and Engineering sciences	0.49 (0.35–0.69) **	0.52 (0.37–0.73) **
Natural and Formal sciences	0.24 (0.16–0.37) **	0.25 (0.16–0.38) **
BMI		
Normal	Reference	Reference
Underweight	1.24 (0.73–2.09)	1.18 (0.68–2.04)
Overweight	0.60 (0.41–0.88) *	0.69 (0.46–1.06)
Obese	0.48 (0.21–1.09)	0.77 (0.32–1.82)
Smoking		
Non-smoker	Reference	Reference
Current smoker	0.69 (0.48–0.98) *	0.75 (0.51–1.11)
Occasional smoker	1.06 (0.68–1.63)	1.10 (0.69–1.75)
Ex-smoker	1.07 (0.56–2.02)	1.17 (0.60–2.29)
Eating behavior		
Regular	Reference	Reference
Irregular meals	0.64 (0.43–0.95) *	0.64 (0.42–0.99) *
Occasionally regular meals	0.60 (0.42–0.85) *	0.64 (0.44–0.92) *

Abbreviations: cOR—crude odds ratio; aOR—adjusted odds ratio; CI—confidence interval; BMI—body mass index; * *p* < 0.05; ** *p* < 0.001.

**Table 4:** The most common types of dietary supplements, vitamins, and minerals used among students.

Dietary Supplements	Medical Sciences *n* (%)	Social Sciences and Humanities *n* (%)	Technology and Engineering Sciences *n* (%)	Natural and Formal Sciences *n* (%)	In Total N (%)
Types of dietary supplements ^†^					
Vitamins	148 (25.4)	60 (28.6)	80 (29.1)	39 (30.9)	327 (27.4)
Minerals	115 (19.8)	46 (21.9)	65 (23.6)	28 (22.2)	254 (21.3)
Combination of vitamins and minerals	134 (23.0)	32 (15.2)	41 (14.9)	21 (16.7)	228 (19.1)
Probiotics	65 (11.2)	15 (7.1)	24 (8.7)	12 (9.5)	116 (9.7)
Proteins and Amino acids	40 (6.7)	21 (10.0)	26 (9.5)	9 (7.1)	96 (8.1)
Fish oil supplements	32 (5.5)	14 (6.7)	18 (6.6)	9 (7.1)	73 (6.1)
Herbal supplements	40 (6.7)	14 (6.7)	11 (4.0)	5 (3.9)	70 (5.9)
Creatine	8 (1.4)	8 (3.8)	10 (3.6)	3 (2.4)	29 (2.4)
Vitamins ^†^					
A	19 (4.2)	4 (2.9)	7 (3.3)	2 (1.7)	32 (3.4)
D	33 (7.2)	8 (5.7)	15 (7.1)	12 (10.0)	68 (7.3)
E	19 (4.2)	7 (5.0)	8 (3.8)	3 (2.5)	37 (3.9)
C	139 (30.4)	37 (26.4)	63 (29.7)	27 (22.5)	266 (28.6)
B6 (Pyridoxine)	21 (4.6)	14 (10.0)	10 (4.7)	10 (8.3)	55 (5.9)
B7 (Biotin)	11 (2.4)	0 (0.0)	4 (1.9)	4 (3.3)	19 (2.1)
B9 (Folic acid)	24 (5.3)	2 (1.4)	6 (2.8)	8 (6.7)	40 (4.3)
B12 (Cyanocobalamin)	24 (5.3)	12 (8.6)	12 (5.7)	8 (6.7)	56 (6.0)
B complex	92 (20.1)	29 (20.7)	47 (22.2)	28 (23.3)	196 (21.1)
Multivitamins	71 (15.5)	26 (18.6)	40 (18.9)	18 (15.0)	155 (16.7)
Other	4 (0.9)	1 (0.7)	-	-	5 (0.5)
Minerals ^†^					
Magnesium	143 (40.4)	47 (46.1)	67 (36.0)	36 (36.7)	293 (39.6)
Zinc	86 (24.3)	16 (15.7)	39 (20.9)	21 (21.4)	162 (21.9)
Calcium	32 (9.0)	9 (8.8)	21 (11.3)	14 (14.3)	76 (10.3)
Iron	26 (7.3)	13 (12.8)	21 (11.3)	12 (12.2)	72 (9.7)
Selenium	28 (7.9)	5 (4.9)	22 (11.8)	9 (9.2)	64 (8.7)
Chromium	6 (1.7)	0 (0.00)	4 (2.2)	2 (2.0)	12 (1.6)
Minerals combination	32 (9.0)	12 (11.8)	12 (6.5)	3 (3.1)	59 (7.9)
Other	1 (0.3)	-	-	1 (1.0)	2 (0.3)

^†^ No statistically significant differences in frequencies among students of different academic fields (Chi-square test).

**Table 5:** Practice and students’ knowledge related to dietary supplements use.

Variables	Medical Sciences *n* (%)	Social Sciences and Humanities *n* (%)	Engineering and Technology Sciences *n* (%)	Natural and Formal Sciences *n* (%)	Dietary Supplement Users (%)
Number of dietary supplements					
1	84 (32.2)	30 (34.1)	45 (38.8)	11 (25.0)	170 (33.4)
2	92 (35.2)	30 (34.1)	28 (24.1)	23 (52.3)	173 (34.0)
3	47 (18.0)	12 (13.6)	21 (18.1)	4 (9.1)	84 (16.5)
≥4	38 (14.6)	16 (18.2)	22 (18.9)	6 (13.6)	82 (16.1)
Form of dietary supplements					
Tablets	163 (37.6)	49 (32.0)	74 (33.9)	25 (33.3)	311 (35.4)
Effervescent tablets	91 (21.0)	26 (17.0)	43 (19.7)	19 (25.3)	179 (20.4)
Capsules	73 (16.9)	29 (19.0)	34 (15.6)	12 (16.0)	148 (16.8)
Powder	90 (20.8)	37 (24.2)	54 (24.8)	16 (21.3)	197 (22.4)
Liquids	12 (2.8)	8 (5.2)	8 (3.7)	2 (2.7)	30 (3.4)
Other	4 (0.9)	4 (2.6)	5 (2.3)	1 (1.3)	14 (1.6)
Place of purchase ***					
Pharmacy	247 (85.5)	67 (63.8)	99 (75.0)	41 (80.4)	454 (78.7)
Supermarket/grocery store	22 (7.6)	15 (14.3)	11 (8.3)	8 (15.7)	56 (9.7)
Fitness center	12 (4.2)	9 (8.6)	14 (10.6)	2 (3.9)	37 (6.4)
Other	8 (2.7)	14 (13.3)	8 (6.1)	0 (0)	30 (5.2)
Source of recommendation					
Doctor	88 (26.2)	40 (31.7)	43 (23.4)	18 (28.6)	189 (26.7)
Pharmacist **	108 (32.1)	15 (11.9)	38 (20.7)	17 (26.9)	178 (25.1)
Nutritionist	13 (3.9)	2 (1.6)	8 (4.3)	3 (4.8)	26 (3.7)
Fitness trainer	22 (6.5)	11 (8.7)	17 (9.2)	3 (4.8)	53 (7.5)
Friend ***	38 (11.3)	30 (23.8)	33 (17.9)	16 (25.4)	117 (16.5)
Media, Internet **	42 (12.5)	23 (18.3)	36 (19.6)	5 (7.9)	106 (14.9)
Other	25 (7.4)	5 (4.0)	9 (4.9)	1 (1.6)	40 (5.6)
Use during a season					
Spring	221 (45.9)	79 (47.3)	93 (42.9)	29 (40.2)	422 (45.0)
Summer	18 (3.7)	12 (7.2)	17 (7.8)	3 (4.2)	50 (5.3)
Autumn	46 (9.5)	15 (9.0)	37 (17.0)	8 (11.1)	106 (11.3)
Winter	197 (40.9)	61 (36.5)	70 (32.3)	32 (44.4)	360 (38.4)
Self-reported knowledge on dietary supplements ***					
Very good	49 (22.7)	9 (10.2)	18 (15.5)	8 (18.2)	84 (16.5)
Good	113 (43.3)	25 (28.4)	36 (31.0)	5 (11.4)	179 (35.2)
Enough	73 (27.9)	32 (36.4)	37 (31.9)	20 (45.5)	162 (31.8)
Not enough	26 (9.9)	22 (25.0)	25 (21.6)	11 (25.0)	84 (16.5)
Beneficial effects					
Always	86 (32.9)	29 (32.9)	38 (32.8)	16 (36.4)	169 (33.2)
Sometimes	125 (47.9)	41 (46.6)	52 (44.8)	18 (40.9)	235 (46.2)
Never	3 (1.1)	0 (0)	2 (1.72)	1 (2.3)	6 (1.8)
Unknown	47 (18.0)	18 (20.5)	24 (20.7)	9 (20.5)	44 (18.6)
Experience of side effects					
Yes	12 (4.6)	4 (4.5)	5 (4.3)	2 (4.5)	23 (4.5)
No	249 (95.4)	84 (95.5)	111 (95.7)	42 (95.5)	486 (95.5)

** *p* < 0.01; *** *p* < 0.001.

## Data Availability

The data presented in this study are available on request.
